# Gain of 20q11.21 in human pluripotent stem cells enhances differentiation to retinal pigment epithelium

**DOI:** 10.1186/s13287-025-04196-7

**Published:** 2025-02-21

**Authors:** Loriana Vitillo, Fabiha Anjum, Zoe Hewitt, Owen Laing, Nidaa A. Ababneh, Duncan Baker, Ivana Barbaric, Peter J. Coffey

**Affiliations:** 1 Rescue, Repair and Regeneration, Institute of Ophthalmology, University College London, London, EC1V 9EL UK; 2 Centre for Stem Cell Biology, School of Biosciences, The University of Sheffield, Sheffield, S10 2TN UK; 3 Cell Therapy Center, The University of Jordan, Amman, Jordan; 4 Centre for Stem Cell Biology and Engineering, University of California Santa Barbara, Santa Barbara, CA USA; 5 NIHR Biomedical Research Centre at Moorfields Eye Hospital NHS Foundation Trust, UCL Institute of Ophthalmology, London, UK; 6 Sheffield Diagnostic Genetic Services, Sheffield Children’s Hospital, Sheffield, S10 2TH UK

**Keywords:** Age-related macular degeneration, Retinal pigment epithelium, Pluripotent stem cells, Copy number variant, BCL-XL, Tumorigenicity

## Abstract

**Background:**

Cell therapies based on human pluripotent stem cells (hPSCs) are in clinical trials with the aim of restoring vision in people with age-related macular degeneration. The final cell therapy product consists of retinal pigment epithelium (RPE) cells differentiated from hPSCs. However, hPSCs recurrently acquire genetic abnormalities that give them an advantage in culture with unknown effects to the clinically-relevant cell progeny. One of the most common genetic abnormalities in hPSCs is the sub-karyotype 20q11.21 copy number variant, known to carry oncogenes. Understanding the impact of this variant on RPE differentiation and its potential for malignant transformation is crucial for the development of safe and effective cell therapies.

**Methods:**

We monitored the RPE differentiation efficiency of hPSCs with or without the 20q11.21 variant. We then phenotyped the purified RPE cells for functionality, purity and tumorigenicity potential.

**Results:**

We observed that 20q11.21 clones exhibited an enhanced differentiation capacity, developing pigmented foci at a higher rate and yield compared to normal clones. Gene expression analysis confirmed the upregulation of key RPE markers in 20q11.21 clones. The enhanced differentiation capacity of 20q11.21 clones was found to be dependent on the activity of BCL-XL, located within the amplicon. Furthermore, we demonstrated that 20q11.21-containing RPE cells displayed a mature phenotype, maintained long-term stability, and exhibited no malignant transformation capacity in vitro.

**Conclusion:**

We demonstrated that gain of 20q11.21 enhances the speed and yield of RPE differentiation without compromising the phenotype of the derivatives. Finally, we discovered that 20q11.21-localised BCL-XL is important for RPE differentiation with potential non-canonical roles in retinal biology.

**Supplementary Information:**

The online version contains supplementary material available at 10.1186/s13287-025-04196-7.

## Background

Age-related macular degeneration (AMD) is a currently incurable leading cause of blindness worldwide, affecting millions of people in the elderly population [1]. Although its causes are complex, AMD arises from the degeneration of retinal pigment epithelium (RPE) cells, which are critical for maintaining retinal function and visual health [2]. In the last decade, regenerative medicine approaches based on transplantation of healthy RPE cells have shown promising results as a cure for AMD [3].

Human pluripotent stem cells (hPSCs), including human embryonic stem cells (hESCs) and induced pluripotent stem cells, are a valuable and unlimited source for generating functional RPE cells and are in clinical trials around the world [3, 4]. However, during the essential process of cellular expansions, genetic aberrations which provide hPSCs with a selective advantage or culture adaptation frequently arise [5].

The genetic instability of hPSCs is a crucial and timely topic in regenerative medicine [5–8]. Abnormalities of unknown effects, but potentially carrying a tumorigenic risk, have already hampered the progression of human transplantations, with a notable case involving RPE used in an AMD trial [9, 10].

Culture-acquired genetic changes in hPSCs are known to be recurrent and non-random [5]. Indeed, most aberrations involve amplification or losses of specific regions on chromosomes 1, 12, 10, 17, 18, 20 and X [11–13]. Strikingly, one of the most common aberrations, found in around 20% of hPSC lines screened worldwide [12, 14], is a small copy number variation (CNV) at location 20q11.21 on chromosome 20. Notably, this CNV can be missed in routine checks of hPSC karyotypes as it often appears below the resolution of karyotyping by G-banding [11, 12]. Even more strikingly, around 20% of benign and malignant cancers harbour a gain in location 20q11.21 [8].

The cellular mechanisms driving the selective advantage of variant hPSCs include acquisition of pro-survival/ anti-apoptotic properties, changes in cell cycle/proliferation rates, alteration of differentiation potential and combination of the above [6]. While for the majority of recurrent variants the driver gene(s) remain unknown, in the case of the 20q11.21, *BCL2L1* has been identified as the driver gene. *BCL2L1* encodes for anti-apoptotic BCL-XL [15]. Extra copies of BCL-XL provide a powerful selective advantage to hPSCs that are subjected to several environmental pressures (i.e. thawing, expansion, cloning, media changes) leading them to quickly overtake the wild type population [15].

If the drivers of advantage for some variants, such as the 20q11.21, are well documented, the effect of those genetic variants on hPSCs-derivatives remain poorly understood. Similarly, the oncogenic risk of transplantation of pluripotent cells has been the at the centre of attention. We know undifferentiated cells form teratomas in vivo and that many hPSCs acquire oncogenes in culture but we lack information on their specific long-term oncogenic risk in humans. However, little focus has so far been placed on how genetic abnormalities contribute to the tumorigenicity of hPSCs-derived progeny.

Several groups have started to untangle the effects of one of the most common abnormalities, the 20q11.21, on hPSCs differentiation [6]. To date, the evidence regarding 20q11.21 points to altered differentiation potential and tumorigenic risk in the derivatives [8]. It has been shown that 20q.11.21 led to an aberrant neuronal differentiation but the cells were able to produce neural-rosettes and precursors [16]. In SCID mice, these 20q11.21-derived neural precursors produced tumours but not teratomas [16]. Another group found that 20q11.21 reduced, but not ablated, directed neuroectoderm differentiation while having no effect on mesoderm [17]. Moreover, scRNA-seq performed on 20q11.21 embryoid bodies revealed a reduced ectodermal differentiation propensity [18]. Histological teratoma analysis of 20q11.21 highlighted the presence of pigmented cells (i.e. possibly RPE) and stratified squamous epithelium in their ectodermal component [18]. These teratomas were not deemed tumorigenic [18]. In the liver, the 20q11.21 causes a small decrease in the differentiation efficiency of pluripotent cells to hepatocytes, which however showed greater engraftment and formation of tumorigenic lesions than wild type cells [19].

There are no reports on the functional effects of 20q11.21 for the clinically-relevant RPE lineage. This is especially lacking considering that, as mentioned earlier, the presence of genetic variants of unknown effects have already halted clinical trials based on hPSC-derived RPE cells [10]. Thus, it is crucial to understand if and to which extent a safety risk is posed by variants on RPE cells.

We previously characterised a rarer and larger chromosome 20 abnormality, isochromosome 20q, during our clinically-relevant hPSCs-RPE differentiation protocol, but unexpectedly found it prevented progression of spontaneous differentiation and RPE formation tout court [20].

The 20q11.21-localised BCL-XL plays a critical role in maintaining the viability and function of various cell types, including retinal pigment epithelium (RPE) cells [21]. Studies have shown that alterations in BCL-XL expression can influence cellular fate decisions and differentiation outcomes [22]. In the context of RPE differentiation, understanding the role of BCL-XL within the 20q11.21 becomes particularly relevant.

In this study, we aim to address the knowledge gaps concerning the impact of the 20q11.21 on hPSC-RPE differentiation and its potential implications for tumorigenicity. Understanding the impact of 20q11.21 on RPE cell function would aid the risk assessment and translational path of hPSCs-based therapies for AMD.

## Methods

### Cell lines

Derivation of the MasterShef-7 cell line was performed in the Stem Cell Derivation Facility at the Centre for Stem Cell Biology, University of Sheffield, under HFEA licence R115-8-A (Centre 0191) and HTA licence 22,510, in a clean room setting, following strict standard operating procedures. Clonal lines were generated as described previously [20]. Derivation of the hIPSCs JUCTCi010-A, B and C cell lines were performed in the iPSC lab at the Cell Therapy Center, University of Jordan, as described previously [23].

### Karyotyping and FISH

Karyotyping and FISH were performed by Sheffield Diagnostic Genetic Services (Sheffield Children’s Hospital, UK). For karyotyping, cells were arrested in metaphase with KaryoMAX Colcemid (Thermo Fisher Scientific) followed by a methanol-based fixative method as previously described [24].

Biologically independent hPSCs JUCTCi010-A, B and C cell lines giving rise to RPE 11, RPE 12, RPE 6 were assessed for common karyotype abnormalities with the hPSC Genetic Analysis kit following manufacturer instructions (07550, Stem Cell Technologies).

### Genetic copy number q-PCR assay

Quantitative PCR detection of copy number at loci 1p, 12p, 17q, 18q and 20q was performed with our previously described method [11, 25]. Length of chromosome 20q11.21 was detected with UPL probes as previously described using RELL1 as reference locus gene [24].

### Cell culture

Undifferentiated hESCs were cultured on Nutristem hPSC XF medium (Biological Industries) on Laminin 521 (Biolamina) coated wells. HESCs were passaged with 0.5 mM EDTA (Invitrogen) every 5 days and frozen in Bambanker (Anachem, BB03).

### hESC-RPE differentiation

HESCs were passaged with EDTA and re-plated onto 6-well plates coated with Matrix Matrigel hESc qualified (Corning, 734–1440) in Nutristem medium. Colonies were grown until confluence with daily media change. Differentiation to RPE was initiated by transitioning cultured to differentiation media TLP consisting of Knockout DMEM, 20% Knockout serum replacement, 1% Non-essential amino acids, 1 mM L glutamine and 0.1 mM β-mercaptoethanol (Invitrogen, Life Technologies) as previously described [26, 27]. TLP was replaced every week day for the first 14 days of differentiation followed by a twice-weekly regime.

Pigmented foci were imaged via a customised Epson V7500 flatbed scanner as previously described [28]. Images were converted to grayscale before batch analysis on ImageJ software with a custom Macro that apply the analyse particles function on defined circle areas at the centre of the wells (to avoid background shadow interference at the edges). Summary of percentage of area counts from the replicate wells of each plate were exported and plotted.

Cells were allowed to spontaneously differentiated for 9 weeks at which point pigmented RPE foci, visible by the naked eye, were manually dissected using a microblade (Interfocus). Purified foci were washed with PBS^−/−^ and resuspended into Accutase (Sigma) at 37 °C for 2–3 h until foci appear to have dissociated into a cell dissociation. The RPE cell suspension was further filtered through a 70 μm cell strainer (Corning). Filtered RPE were seeded onto Matrigel-coated 48-well plates at a density of 4.8 × 10^4^ cells/well. RPE were grown and maintained long-term as a confluent monolayer in a twice-weekly TLP medium regimen. Long-term cultures and passaged RPE were stably grown in X-Vivo medium (Lonza) in a twice-weekly feeding regime. Characterisation by immunofluorescence and metabolic activity was assessed in 8 weeks + RPE monolayers.

### Immunofluorescence

Cells were fixed at room temperature for 30 min in 4% paraformaldehyde followed by three washes in DPBS. Blocking and permeabilization was performed in DPBS plus 5% Normal Donkey Serum (Jackson Immuno Research Laboratories) in 0.3% triton X-100 (Sigma). Cells were incubated overnight at 4 °C with primary antibodies diluted in 1% donkey serum, 0.3% triton X-100. Next, cells were washed 3 times with DPBS and incubated for 2 h at room temperature with fluorescent FITC- or TRITC-conjugated donkey secondary antibodies against IgG species matching (Jackson ImmunoResearch Laboratories) at a dilution of 1:100 in 2% donkey serum, 0–3% triton X-100 (in DPBS). Cells were washed 3 times with DPBS. For nuclei counterstain, cells were incubated for 60 s with DAPI (Sigma, diluted 1:2500) followed by another 3 washes in DPBS. Primary antibodies and dilutions: mouse anti PMEL17 (Dako, Mo634, 1:50); mouse anti CRALBP (Invitrogen, MA1-813, 1:1000); goat anti OTX2 (Santa Cruz, 30,659, 1:100); mouse anti PEDF clone 10F12.2 (Millipore, MAB1059, 1:1000); mouse anti TGFB1 (Santa Cruz, 3C11, 1:500), rabbit anti OCT-4 (Cell signalling, C30A3, 1:400). Imaged were acquired using a EVOS FL microscope (Life Technologies) and analysed using Cell Profiler.

### Real-time quantitative PCR

RNA was extracted from cell pellets using the PureLink RNA mini kit following manufacturer's instruction (Invitrogen). RNA was retrotranscribed to cDNA using the SuperScript III Synthesis System (Invitrogen) or the High capacity cDNA Reverse transcription kit (Applied Biosystems). Transcript levels were detected using PowerSYBR® Green master mix (Applied Byosystems) with StepOne Plus (Applied Biosystems). Gene expression was normalised to the housekeeping gene GAPDH and calculated using the 2^−ΔΔCt^ algorithm. Primers sequence for PMEL17 (F: GAT GGC AGG TTA TCT GGG TCA AC; R: GCT GGA ATG AGC AAG AGG CAC ATA G);

Lin28 (F: AATTAGCCGGGTGTGGTGGT; R: GCCTCCTGACCCCACTTTCT).

### PEDF ELISA assay

Presence of the PEDF antigen in the supernatant of RPE cultures was detected using the Human PEDF ELISA Assay (PED613-Uman, XpressBio) following manufacturer instruction. Optical density was read at 450 nm on a Tecan microplate reader.

### Small molecules and morphogens

BCL-xL inhibitor A1155463 (Cayman chemicals, 27,369), Z-VAD (Ome)-FMK (Cell signalling, 60,332).

### Live caspase-3 assay

Cells were plated at 2,000 cells/well in TLP medium on 96-well microplates coated with Matrigel. After 48 h from seeding medium was replaced with fresh TLP supplemented with 1M NuncView®488 Caspase-3 substrate (Biotium). After 30’ of incubation at room temperature the first t0 time-point was recorded on an EVOS microscope fitted with fluorescence filters (Life Technologies). Live caspase-3 signal intensity was thereafter imaged in the same wells at t8 and t24 hours. In between time-points cells were incubated at 37 °C 5% CO_2._

### Soft agar colony formation assay

Soft Agar Colony Formation was performed using the CytoSelect™ 96-well Cell Transformation Assay following manufacturer’s instructions (CBA-130-T, Cell Biolabs). Cells were plated at a concentration of 2 × 10^3^ cells/well in 1.2% soft agar. Cells were cultured and monitored for 35 days for the presence of colonies and imaged with a standard phase microscope. HeLa cancer cell lines were used as control at the same concentration (courtesy gift of Loise Wong, Institute of Ophthalmology, UCL).

### Statistical analysis and reproducibility

Statistical analysis was performed on triplicate independent experiments with GraphPad prism with *p* < 0.05 defining statistical significance. Images are shown as the representative of all independent experiments.

## Results

1. Gain of the 20q11.21 amplicon enhances speed and yield of RPE differentiation

**Fig. 1:**
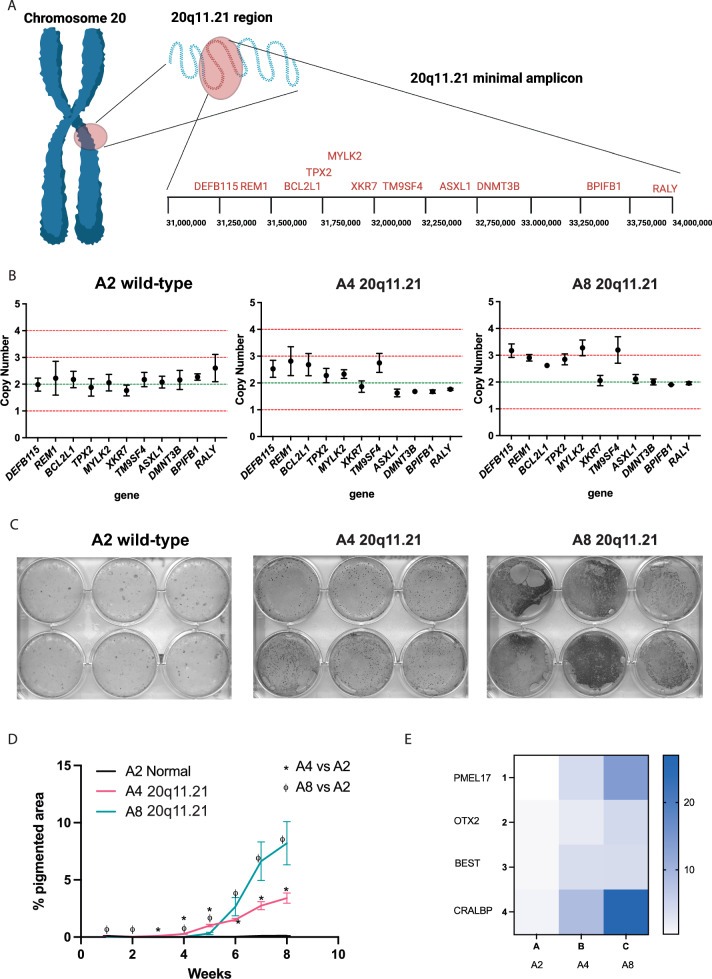
**A** Diagram of 20q11.21 region position of key genes localised within the minimal amplicon. **B** qPCR copy number assay of genes in the 20q11.21 region showing the size and breakpoint of the amplicon in A4 and A8 clonal lines compared to A2 wild-type control. **C** Representative pigmented area at week 8. **D** Quantification of pigmented area from scan imaging over the 8 weeks of RPE differentiation in 20q11.21 clones A4 and A8 versus normal A2 cells*. **E** Heat map of qPCR gene expression levels of RPE pigmentation markers in bulk sampling of 20q11.21 vs wild-type control at week 9 of RPE differentiation. Values plotted are from 2^−ΔΔCt^ compared to wild-type control. *The data shown in (D) are presented as mean plus SEM of three independent experiments, and statistical significance was determined using Student’s t test, two-tailed, *p* ≤ 0.05

We previously isolated hESCs which had acquired in culture the common sub-karyotype abnormality of 20q11.21 (Fig. 1A) and banked clonal isogenic wild-type subline (A2) and two 20q11.21 sublines (A4 and A8) [20]. We qualified the clonal lines with our rapid qPCR assay [24] to determine the length of the 20q11.21, finding the amplicon breakpoint after the TM9SF4 locus (Fig. 1B, Additional Figure S1 A).

We set forth to test how 20q11.21-carrying hESC performed in our RPE differentiation protocol compared to their normal isogenic counterparts. (Fig. 1C). Strikingly, we observed that 20q11.21 clones developed pigmented foci visible by the naked eye at a higher quantity than normal clones (Fig. 1C). Accordingly, quantification of pigmented areas via scanner imagining showed that over the weeks of spontaneous differentiation the 20q11.21 clones develop RPE foci at a significantly faster rate and to higher density than normal clones (Fig. 1D). Furthermore we observed a degree of variability in the RPE yields between the stem cell clones, a phenomenon which is often due to smaller genetic/epigenetic variations even between isogenic lines [7]. Notably, 20q11.21-carrying cells have a high RPE yield not only compared to their own normal isogenic counterpart but also if compared to other hPSCs lines (human induced pluripotent stem cells) which have tested normal for the most common genetic abnormalities [29], including on chromosome 20q, by qPCR assay (Additional Figure S1 B–C).

In line with the imaging data, gene expression analysis showed that 20q11.21 clones had higher expression of the key markers of RPE cells, such as pre-melanosome protein PMEL17, CRALBP, OTX2 and BEST, compared to wild-type clones (Fig. 1E). Overall, our data show that 20q11.21 clones have boosted speed and yield of RPE differentiation compared to their wild-type counterparts.

2) 20q11.21 RPE are phenotypically normal

We next asked whether *bona fide* RPE can be isolated from 20q11.21 pigmented foci despite their abnormal genetic makeup. Normal clones, in view of their low yield, were not used to isolate RPE.

**Fig. 2:**
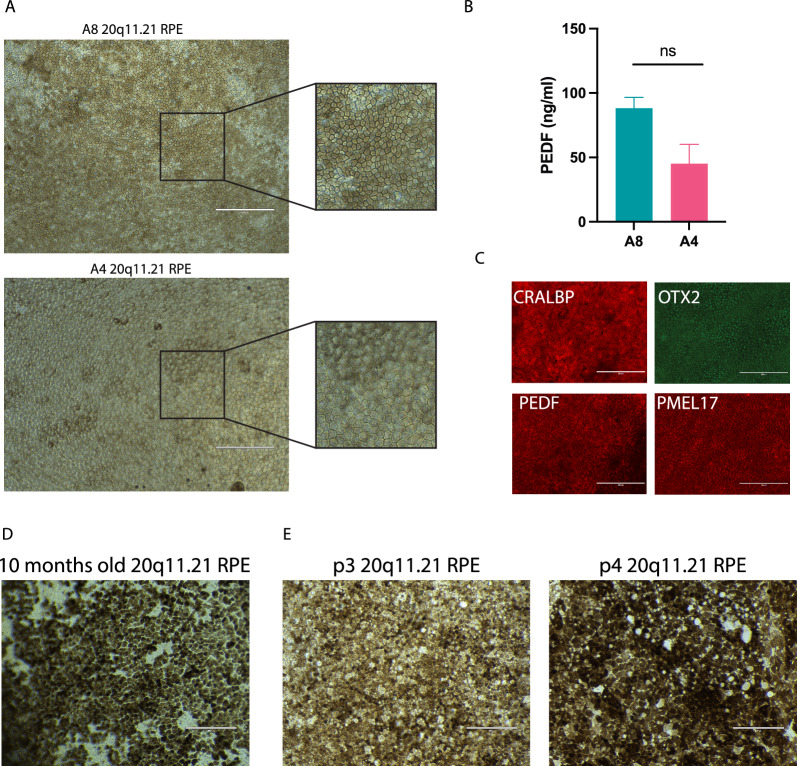
**A** Representative phase images of 20q11.21 RPE monolayer and cobblestone morphology (insert). **B** PEDF concentration in 2 months-old 20q11.21 RPE monolayers*. **C** Immunofluorescence images showing positivity for RPE markers CRALBP, OTX2, PEDEF, PMEL17 and negativity for OCT4 and TGFβ in A8 20q11.21 RPE. **D**–**E** Representative phase images of 20q11.21 RPE monolayer cobblestone morphology at 10 months from derivation in passage 0 and passage 3 and 4. *The data shown in (B) are presented as mean plus SEM of three independent experiments, and statistical significance was determined using Student’s t test, two-tailed. ns = not significant

Purified 20q11.21 RPE matured the cobblestone morphology and pigmentation typical of an RPE monolayer (Fig. 2A). Moreover, they were able to secrete Pigment Epithelium Derived Factor (PEDF), a hallmark of healthy RPE cells, showing that they are metabolically active (Fig. 2B). The long-term stability of the RPE monolayer is of fundamental importance for cell therapy due to prolonged manufacturing and for the functional survival of the transplant in patients. We validated their phenotype after 6 months of continuous culture (thought to be a cut-off point for RPE destined for transplantation), finding that they ubiquitously expressed characteristic markers CRALBP, OTX2, PEDF and PMEL17 (Fig. 2C, Additional Figure S2 A). Moreover, we found that 20q11.21 RPE could be maintained even longer without losing pigmentation, with the longest time assessed being 10 months (Fig. 2D). Importantly, we observed no abnormal proliferation (multilayer overgrowth of cells) at 6 months or even 10 months of culture, shown by the persistence of a distinct RPE monolayer (Fig. 2C–D).Finally, we found that 20q11.21 RPE could be passaged up to passage 4 but the cells lost morphological attributes becoming larger and displaying white intracellular areas (possibly lipid deposits), a phenomenon recorded also in normal RPE (Fig. 2E). In summary, 20q11.21 RPE cells develop into a typical monolayer with long-term stable morphology, markers and metabolic activity.

3) BCL-XL activity drives the RPE differentiation boost of 20q11.21 variants

To investigate the potential mechanism responsible for the 20q11.21 variant enhanced pigmentation, we tested the main genetic driver of the variant growth advantage, *BCL2L1* (which encodes for BCL-XL), which is located within the amplified region [15]. Importantly, primary human RPE and the ARPE-19 line express high levels of BCL-XL shown to be critical for their resistance to cell death, but there are no reports in the context of hPSC-RPE differentiation [21].

**Fig. 3:**
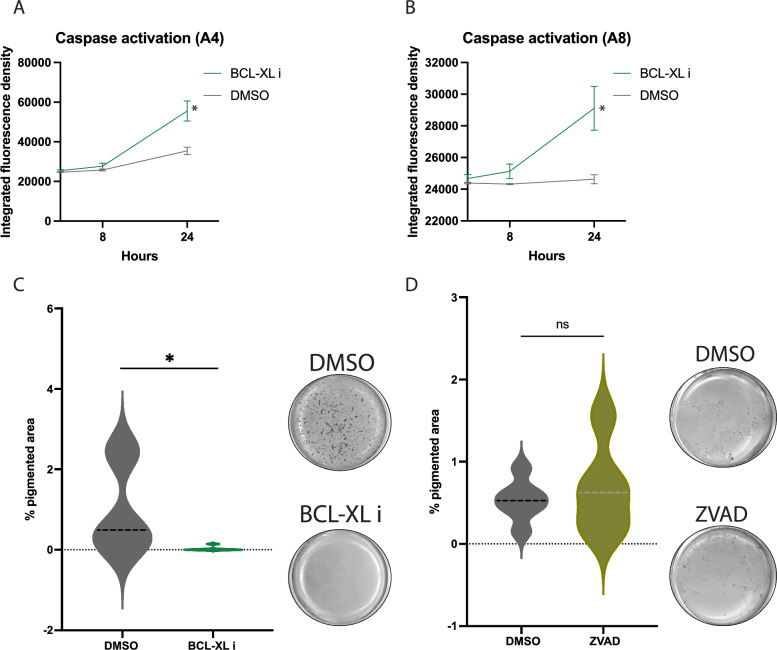
**A–B** Caspase activation using live imaging for 24 h in (A) A4 and (B) A8 20q11.21 variants treated with DMSO control or 70 nM BCL-xL i *. **C** Violin plot of quantification of pigmented area from scan imaging over the 9 weeks of RPE differentiation in A8, A4 20q11.21 variants treated with DMSO control or 70 nM BCL-xL i and corresponding representative images *. **D** Violin plot of quantification of pigmented area from scan imaging over the 9 weeks of RPE differentiation in wild-type cells treated with DMSO control or (10 μM) ZVAD-FMK and corresponding representative images *. *The data shown in (A)–(B) are presented as mean plus SEM while in (C)–(D) as violin plot of three independent experiments, and statistical significance was determined using Student’s t test, two-tailed. *p ≤ 0.05; ns not significant

We hypothesised that BCL-XL plays an important role in the emergence and pigmentation yield of 20q11.21 variants. To this end, we selectively inhibited BCL-XL during the whole RPE differentiation protocol with a potent and selective BH3 mimetic ligand, A1155463 (from here renamed BCL-XLi) [30]. BCL-XL exerts its main canonical role as an anti-apoptotic protein at the mitochondria [31]. Thus, as a read-out of effective inhibition in our model, we measured cleaved-caspase 3 activity for the first 24 h of the RPE differentiation of 20q11.21 variants. We employed a live cell imaging assay based on the caspase-activated DEVD-NuncView488 fluorogenic substrate, which is non-toxic and does not interfere with the progression of apoptosis [32]. This resulted in a progressive increase of caspase 3 activity in both 20q11.21 variant pairs at the tested low concentration of 70 nM (corresponding to the EC_50_ of A1155463 in H146 lung cancer cells) (Fig. 3A–B). Next, we treated the 20q11.21 variants with 70 nM of BCL-XLi at every media change during the RPE differentiation (Bi-weekly after the first two weeks). We found that 9 weeks of inhibition of BCL-XL had dramatically reduced the foci yield compared to the DMSO control, without killing the cells (observational data, not shown), effectively dialling down the boost on RPE differentiation that we observed in 20q11.21 variants (Fig. 3C).

BCL-XL is a key regulator of cell survival, localising at the mitochondria to prevent cytochrome C release and a caspase-dependent apoptosis via interaction with other BCL-2 family members [33, 34]. However, beyond its well-established function in preventing apoptosis, there is increasing evidence of BCL-XL non-apoptotic roles which include metabolism, mitochondrial dynamics and calcium homeostasis [33, 34]. Other studies have revealed that BCL-XL regulates cell fate determination, for example by impairing neuroectodermal differentiation [25]. Therefore, we asked whether BCL-XL antiapoptotic effect is the main mechanism for the increased RPE yield of 20q11.21 variants. To address this question, we rendered the isogenic normal control cells more resistant to cell death during RPE differentiation by treatment with the irreversible caspase-inhibitor ZVAD-FMK (5 µM for the first 3 days followed by 10 µM at each media change). We found that ZVAD-FMK had not significantly increased the pigmentation of normal cells compared to control, suggesting that BCL-XL enhanced the RPE yield via alternative non-apoptotic pathways (Fig. 3D). Further studies would be required to evaluate the full mechanism of BCL-XL in RPE early differentiation. For example, overexpression of BCL-XL in hPSCs up to and above 3 copies, such as in 20q11.21, could help dissociate its scaffolding functions to BH3-dependent roles. Taken together, these results illustrate that BCL-XL underpins the enhanced pigmentation of 20q11.21 variants as is required for hPSCs-RPE differentiation. Finally, this process is likely executed by BCL-XL non-apoptotic mechanisms, a prospect which opens new areas of investigation in eye research and retinal biology.

4) 20q11.21 RPE display a low tumorigenic risk in vitro

**Fig. 4:**
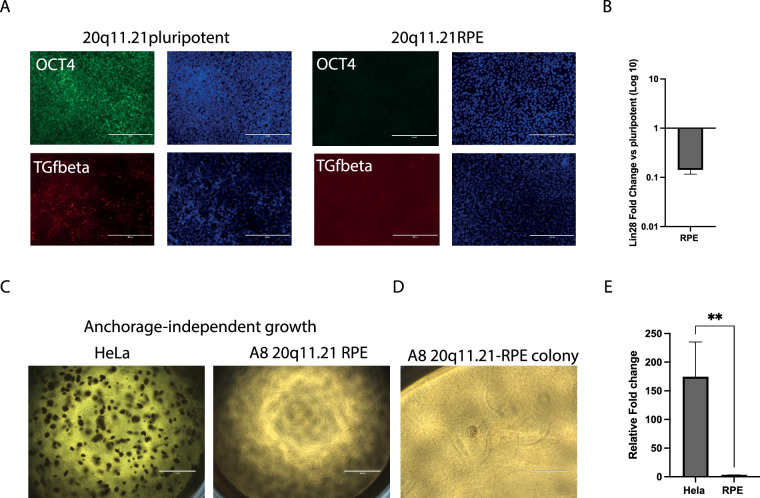
**A** Immunofluorescence images showing positivity for pluripotency marker OCT4 and active TGFβ in pluripotent A8 20q11.21 hESCs. Immunofluorescence images showing negativity for pluripotency marker OCT4 and associated TGFβ in A8 20q11.21 RPE. **B** Gene expression levels of pluripotency marker Lin28 in 20q11.21 RPE cells. **C** Anchorage-independent growth assay in A8 20q11.21 RPE versus cancer cell line HeLa at 35 days. **D** Insert from data in (C) showing only visible group of cells in 20q11.21 RPE sample. **E** Quantification via colony count of anchorage-independent assay shown in (C); data are presented as mean plus SEM and statistical significance was determined using Student’s t test, two-tailed, **p = 0.008

A key concern for the safe use of hPSCs in cell therapy is the potential for malignant transformation of the transplants. Firstly, pluripotent cells have the known potential to form teratomas, especially if harbouring the 20q11.21 amplicon [25]. Secondly, a hPSCs-progeny carrying the 20q.11.21 could be primed for tumorigenicity due to the presence of oncogenes in the locus and the known association of this genetic variant with several cancers [8]. However, the 20q11.21 tumorigenic risk is unknown for the RPE lineage. Firstly, we found no trace of pluripotency-associated marker OCT4, and TGF-beta, both expressed at the pluripotent stage, in 6-months old 20q11.21 RPE by immunocytochemistry (Fig. 4A, Additional Figure S3A–B). To further look for traces of residual pluripotent cells we quantified the Lin28 pluripotency marker by qPCR, proven to be a highly sensitive method in RPE [35]. Others showed that Lin28 mRNA is above detectable levels in hPSCs-derived RPE qualified for clinical trials which included primary RPE controls [9]. We found that 20q11.21 RPE cells have significantly downregulated Lin28 expression compared to undifferentiated cells (Fig. 4B). Combined, our data on 20q11.21 RPE show no contamination with pluripotent cells, although we cannot categorically say this to be zero.

Beyond expression of pluripotency markers, a reliable test of the risk posed by 20q11.21 in the RPE is the functional assessment of their potential for malignant transformation. To this end, we performed a robust tumorigenicity in vitro assay based on soft-agar colony formation on 20q11.21 RPE. The use of soft-agar as a semi-solid matrix allows cells to grow in a manner that resembles their growth in a solid tumour. This assay enables the assessment of anchorage-independent growth, a hallmark of tumorigenicity. The main advantage of the soft agar tumorigenicity assay is its ability to discriminate between transformed or tumorigenic cells and non-transformed or non-tumorigenic cells. Transformed cells, which have acquired the ability to form tumours, can proliferate and survive in the soft agar, forming visible colonies. In contrast, non-transformed cells typically fail to grow or form only small colonies in the soft agar. Furthermore, by monitoring the growth and morphology of colonies over an extended period, the assay provides insights into the sustained proliferative capacity and clonogenic potential of cells. This information is crucial for assessing the tumorigenicity of cells and predicting their behaviour in vivo. Finally, the colony formation assay is unable to detect undifferentiated hPSCs due to their inability to survive in single cell conditions [35]. This is an advantage for our experimental condition, as we aimed to primarily test the contribution of the 20q11.21 to the tumorigenicity potential of RPE and not of residual undifferentiated cells. After 35 days in soft-agar, we found one small aggregate in 20q11.21 RPE while control cancer cell line Hela formed many large and proper colonies (Fig. 4C–D). We thus report no significant correlation between the 20q11.21 abnormality and tumorigenicity of RPE derivatives in vitro (Fig. 4E). The lack of anchorage-independent growth of 20q11.21 RPE demonstrates that the overexpression of the BCL2L2 oncogene within the 20q11.21 locus does not result in a high risk of tumorigenicity for the RPE lineage. The presence of one aggregate, even if not in the form of an established growing colony, is a reminder that such risk, however small, is not absent. The use of animal models to further test the tumorigenicity of the 20q11.21 RPE is an obvious next step, but considering the increasing validation of the soft-agar method used here, and in keeping with the principles of 3Rs (Replacement, Reduction, Refinement) in animal research, we consider this unnecessary for the present work. Taken together, our data show that 20q11.21 RPE, despite their abnormal karyotype and survival advantage, have reached stable maturity without carrying large traces of undifferentiated cells behind and present a low risk for malignant transformation as assessed in vitro.

## Discussion

Here we explored the common chromosome 20 abnormality of hPSCs during a clinically relevant hPSCs-RPE differentiation protocol currently in use for the treatment of age-related macular degeneration (AMD) [26]. We discovered that hPSCs carrying the 20q11.21 variant have an enhanced RPE differentiation compared to their normal isogenic counterparts, and that this is dependent on the activity of BCL-XL, located in the amplicon.

BCL-XL is a known oncogene [30] and there are specific concerns regarding the potential for malignant transformation posed by 20q11.21 abnormal cells moving through stem cell therapy processes and transplantation [8, 36, 37]. Currently, there is no international consensus on standardised tumorigenicity assays for pluripotent stem cell derivatives, but several studies propose sensitive in vitro methods to detect undifferentiated and malignant cells in the final cell therapy product [36, 38]. Our results provide the first evidence that RPE derived from one of the most common abnormality of hPSCs appear phenotypically indistinguishable to a healthy RPE monolayer while are reassuringly devoid of undifferentiated cells or a clear capacity for malignant transformation in vitro. On the other hand, it is especially because of such good propensity to generate bona fide RPE, coupled with the known elusiveness to detection by G-banding karyotyping [11], that this 20q11.21 variant grants a high degree of caution. It was previously shown that 20q11.21-derived neural precursors, which showed an aberrant differentiation, could not form teratomas or metastases but maintained a tumorigenic capacity in vivo [16]. Although our 20q11.21 RPE could not form malignant colonies in our in vitro experiments, we do not advise for manufacturing with cells harbouring this variant.

Others have clearly shown that the presence of the 20q11.21 amplicon impairs neuroectoderm differentiation [17]. It is thus surprising to see that the RPE, which is a derivative of the neuroectoderm germ layer, is boosted by the presence of the amplicon. This demonstrates that a negative bias towards an entire lineage (in this case ectoderm) cannot rule out the capacity of 20q11.21 cells to perform well toward a more specific cell type. Such evidence is a warning against generalisations and shows the importance of testing the effects of common genetic abnormalities in each relevant protocols, where possible.

Aside from addressing safety issues relevant to stem cell therapy, our study offers novel observations relevant to RPE biology and pathology. Our results propose a new role for BCL-XL in the emergence of an RPE cell type from a pluripotent stem cell culture. Indeed, when we blocked BCL-XL activity, pigmented RPE foci areas disappeared from the differentiation.

To date, BCL-XL has been linked to the survival of primary RPE tissue and the ARPE19 cell line, where is overexpressed [21]. Despite being essential to RPE, BCL-XL pro-survival activity could lead to detrimental effects over time. Accumulation of senescent RPE cells has been observed in the aging retina and in AMD-affected retinas in mice models and in human eyes [25, 39]. These senescent RPE cells exhibit increased expression of BCL-XL, contributing to their resistance to apoptosis, with consequent compromised tissue homeostasis [39, 40]. Targeting the senescent RPE cells through inhibitors of BCL-XL represents a new potential therapeutic strategy for AMD and other retinal degenerative diseases [39, 40].

However, in our differentiation, we didn’t observe more RPE foci areas by suppressing caspases-driven apoptosis, mimicking the final effectors of pro-survival BCL-XL. This means that at least during differentiation, increasing cell survival is not the main mechanisms driving the emergence of more RPE. Notably, BCL2 family members, including BCL-XL, can play several non- apoptotic roles, many of which are attracting increasing attention for their implications in cancer research and other therapy areas [33]. BCL-XL non-canonical functions are pertinent to RPE biology as they involve calcium homeostasis and mitochondrial function, which is essential for the cellular physiology of a healthy RPE monolayer and is impaired in many diseases of the retina [25]. Therefore, we propose that the BCL-XL enhancement of hPSC-RPE differentiation we report here involves its non-canonical functions, which would be interesting to explore in detail in future eye and stem cell research.

## Conclusions

Understanding the functional consequences of frequent genetic abnormalities of pluripotent cells employed in cell therapy is of fundamental important for the safe development of advanced medicinal products. Here we showed that one of the most common genetic abnormality of hPSCs, the 20q11.21 CNV, is permissive for the production of functional RPE and is in fact enhancing the differentiation in a BCL-XL-dependent manner. Crucially, 20q11.21 RPE are void of malignant transformation capacity in vitro but we do not advise for the use of RPE cell therapy products carrying this CNV. This recommendation takes into consideration several cautionary points: the tumorigenicity of 20q11.21 reported in other systems including cancers [8]; the risk of 20q11.21 being a baseline susceptibility factor for following oncogenic mutations [8]; and finally BCL-XL effect on the senescence of RPE over time [40]. Instead, we recommend testing of the 20q11.21 via rapid qPCR at several passages prior and after differentiation. Furthermore, our study can aid an evidence-based risk assessment in the case that 20q11.21 is detected at crucial, early or late, stages of RPE cell therapy manufacturing.

Finally, our results shed an intriguing new light on the role played by BCL-XL during differentiation, and could in the future lead to new approaches to increase RPE derivation efficiency and understanding of their physiopathology.

## Supplementary Information

Supplementary Material 1: Figure S1. Related to Figure 1. **A** Copy numbers of calibrant genes used for the 20q.11.21 amplicon length qPCR assay. **B** Representative pigmented area of three hiPSC-derived normal RPE after 2 months in culture. **C** Fold change qPCR results of hPSCs lines shown in **B**, alongside extra lines and the control, as assessed for abnormalities with the hPSC Genetic Analysis kit

Supplementary Material 2: Figure S2. Related to Figure 2. **A** Immunofluorescence images showing positivity for RPE markers CRALBP, OTX2, PEDF, PMEL17 in A4 20q11.21 RPE

Supplementary Material 3: Figure S3. Related to Figure 4. **A** Immunofluorescence images showing negativity for pluripotency marker OCT4 and associated TGFβ in A4 20q11.21 RPE. **B** Immunofluorescence images showing positivity for pluripotency marker OCT4 in pluripotent A4 20q11.21 hESCs

## Data Availability

The datasets used and/or analysed during the current study are available from the corresponding author on reasonable request.

## Abbreviations

hPSCs: Human pluripotent stem cells
AMD: Age-related macular degeneration
RPE: Retinal pigment epithelium
CNV: Copy number variant
SCID: Severe combined immunodeficiency disease
