# Supplementary figures and images for: Gain of 20q11.21 in human pluripotent stem cells enhances differentiation to retinal pigment epithelium
Source: Stem Cell Res Ther. 2025 Feb 21;16:82. doi: 10.1186/s13287-025-04196-7 (PMC11846190; doi:10.1186/s13287-025-04196-7)

A

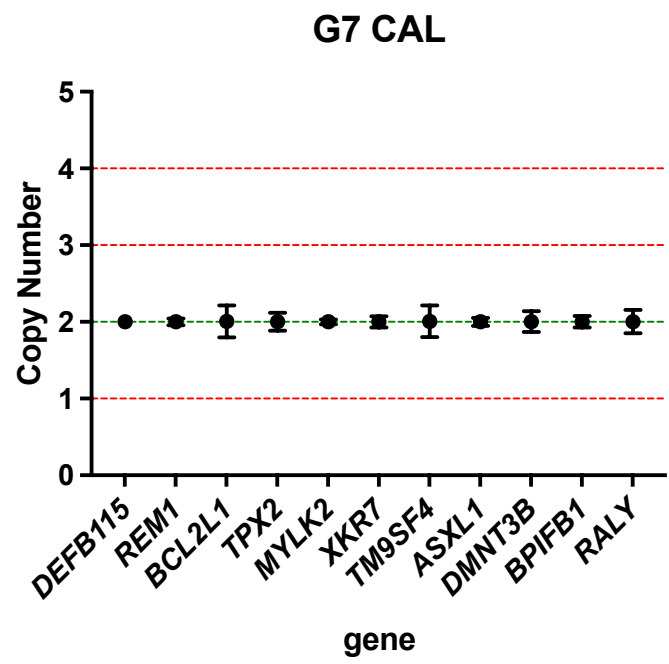

B

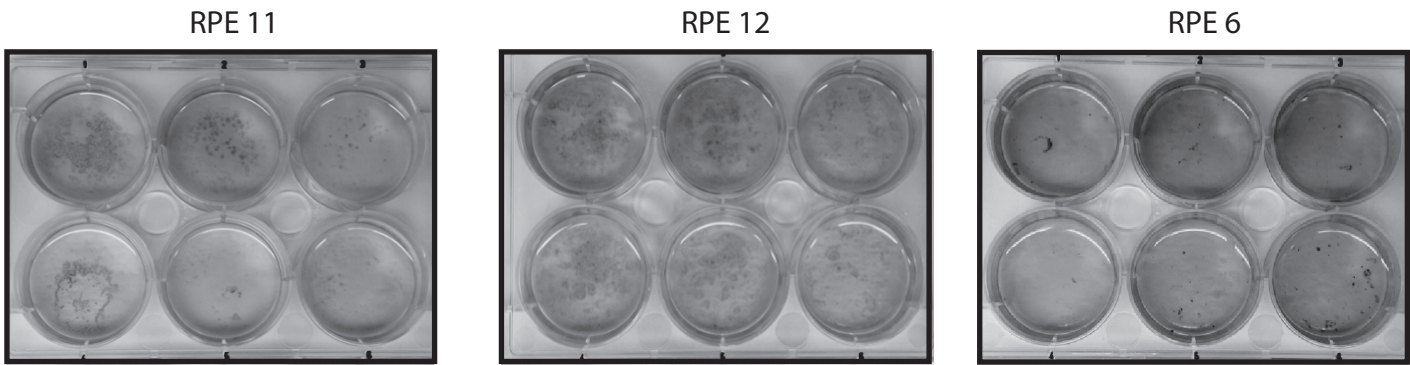

C

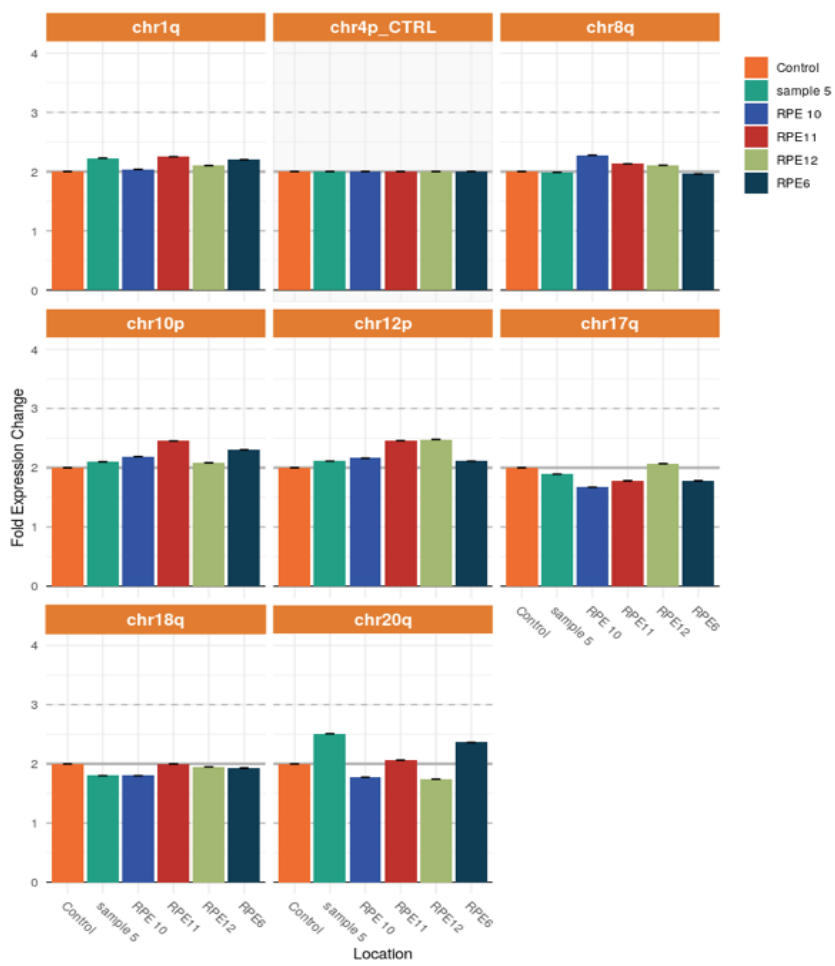

Supplement: Supplementary file 1 — Supplementary Material 1: Figure S1. Related to Figure 1. A Copy numbers of calibrant genes used for the 20q.11.21 amplicon length qPCR assay. B Representative pigmented area of three hiPSC-derived normal RPE after 2 months in culture. C Fold change qPCR results of hPSCs lines shown in B, alongside extra lines and the control, as assessed for abnormalities with the hPSC Genetic Analysis kit [file 13287_2025_4196_MOESM1_ESM.pdf]

A

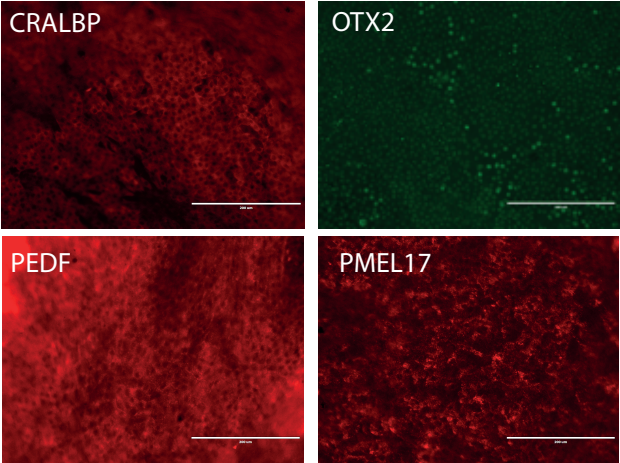

Supplement: Supplementary file 2 — Supplementary Material 2: Figure S2. Related to Figure 2. A Immunofluorescence images showing positivity for RPE markers CRALBP, OTX2, PEDF, PMEL17 in A4 20q11.21 RPE [file 13287_2025_4196_MOESM2_ESM.pdf]

A

20q11.21 RPE

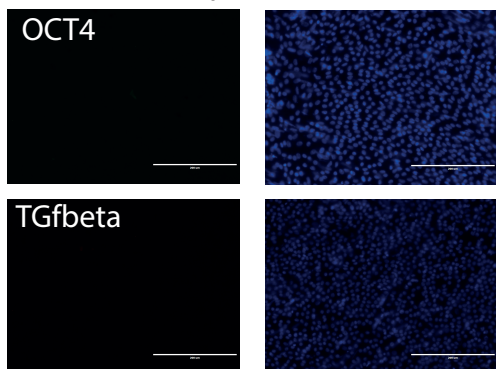

B

20q11.21 pluripotent

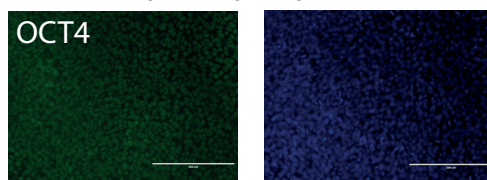

Supplement: Supplementary file 3 — Supplementary Material 3: Figure S3. Related to Figure 4. A Immunofluorescence images showing negativity for pluripotency marker OCT4 and associated TGFβ in A4 20q11.21 RPE. B Immunofluorescence images showing positivity for pluripotency marker OCT4 in pluripotent A4 20q11.21 hESCs [file 13287_2025_4196_MOESM3_ESM.pdf]
